# The best linear unbiased prediction (BLUP) method as a tool to estimate the lifetime risk of pancreatic ductal adenocarcinoma in high-risk individuals with no known pathogenic germline variants

**DOI:** 10.1007/s10689-024-00397-w

**Published:** 2024-05-23

**Authors:** Cristina-Marianini-Rios, María E. Castillo Sanchez, Ana García García de Paredes, Mercedes Rodríguez, Emma Barreto, Jorge Villalón López, Raquel Fuentes, María Muñoz Beltrán, Alfonso Sanjuanbenito, Eduardo Lobo, Alejandra Caminoa, Ignacio Ruz-Caracuel, Sergio López Durán, José Ramón Foruny Olcina, Javier Blázquez, Enrique Vázquez Sequeros, Alfredo Carrato, Jose Carlos Martínez Ávila, Julie Earl

**Affiliations:** 1 Department of Agricultural Economics, Statistics and Business Management, Universidad Politécnica de Madrid, Madrid, Spain; 2 Ramón y Cajal Health Research Institute (IRYCIS), Carretera Colmenar Km 9, 100, Madrid, 28034 Spain; 3 Gastroenterology and Hepatology Department, Hospital Universitario Ramón y Cajal, Madrid, Spain; 4 Centro de Investigación Biomédica en Red de Enfermedades Hepáticas y Digestivas (CIBERehd), Instituto de Salud Carlos III, Madrid, Spain; 5 Medical Oncology Department, Hospital Universitario Ramón y Cajal, IRYCIS, Madrid, 28034 Spain; 6 The Biomedical Research Network in Cancer (CIBERONC), Av. Monforte de Lemos, 3-5. Pabellón 11. Planta 0, Madrid, 28029 Spain; 7 University of Alcalá, Madrid, Spain; 8 Radiology Department, Hospital Universitario Ramón y Cajal, Madrid, Spain; 9 Pancreatic and Biliopancreatic Surgery Unit, Hospital Universitario Ramón y Cajal, Madrid, Spain; 10 Department of Pathology, Hospital Universitario Ramón y Cajal, Madrid, 28034 Spain; 11 Pancreatic Cancer Europe, Brussels, Belgium

**Keywords:** Pancreatic cancer, Genetic cancer risk, Heritability, High-risk screening

## Abstract

Pancreatic ductal adenocarcinoma (PDAC) is the fourth leading cause of cancer-related death in the Western world. The number of diagnosed cases and the mortality rate are almost equal as the majority of patients present with advanced disease at diagnosis. Between 4 and 10% of pancreatic cancer cases have an apparent hereditary background, known as hereditary pancreatic cancer (HPC) and familial pancreatic cancer (FPC), when the genetic basis is unknown. Surveillance of high-risk individuals (HRI) from these families by imaging aims to detect PDAC at an early stage to improve prognosis. However, the genetic basis is unknown in the majority of HRIs, with only around 10–13% of families carrying known pathogenic germline mutations. The aim of this study was to assess an individual’s genetic cancer risk based on sex and personal and family history of cancer. The Best Linear Unbiased Prediction (BLUP) methodology was used to estimate an individual’s predicted risk of developing cancer during their lifetime. The model uses different demographic factors in order to estimate heritability. A reliable estimation of heritability for pancreatic cancer of 0.27 on the liability scale, and 0.07 at the observed data scale as obtained, which is different from zero, indicating a polygenic inheritance pattern of PDAC. BLUP was able to correctly discriminate PDAC cases from healthy individuals and those with other cancer types. Thus, providing an additional tool to assess PDAC risk HRI with an assumed genetic predisposition in the absence of known pathogenic germline mutations.

## Introduction

Pancreatic ductal adenocarcinoma (PDAC) is the fourth leading cause of cancer death in Europe, behind only lung, colon and breast cancer [1, 2]. The 5-year survival rate is less than 10% [3] as the majority of cases have advanced disease at diagnosis, meaning up to 90% of cases have non curable disease [4]. In recent decades, there have been few advances in treatment, with small innovations in neoadjuvant chemotherapy and chemoradiotherapy showing minimal improvements, and surgical resection remains the only potentially curative treatment option [5]. Therefore, there is a clear need for both improved treatment strategies and early detection techniques to increase survival.

The most important non-modifiable risk factor for the development of pancreatic cancer is genetic predisposition. The risk of developing PDAC increases with the number of affected relatives, and the standard HR (Hazard Ratio) is 32 when 3 relatives are affected [6, 7]. Some cases have a hereditary background, with pathogenic germline mutations in cancer risk genes such as BRCA1/2, PALB2, ATM, CHEK2 and CDKN2A [8–11]. Whereas, Familial Pancreatic Cancer (FPC) is defined as families with at least one pair of affected first-degree relatives, with no known genetic basis. This equates to 4–10% of diagnosed cases of PDAC having a familial or hereditary background [12, 13].

PDAC has a relatively low incidence, with 5.7 cases per 100 000 males and 4.1 cases per 100 000 females. This low incidence, combined with the lack of a reliable markers [14], high costs and the limited sensitivity of imaging tests, makes population wide screening impractical. Healthy first- or second-degree relatives of families with hereditary or familial PDAC are the only high-risk population to be offered an imaging based screening program for early detection [15]. The management of high-risk individuals, both in terms of genetic testing and screening, are performed according to European and international guidelines including The International Consortium for Pancreatic Cancer Screening (CAPS) [16], National Comprehensive Cancer Network (NCCN) [17], American Gastroenterological Association (AGA) [18] and American Society for Gastrointestinal Endoscopy (ASGE) [19]. The aim of screening high-risk individuals (HRIs) is early detection of PDAC during a potentially curable stage, as well the detection of pre-malignant precursor lesions, such as intraductal papillary mucinous neoplasms (IPMN) with high-grade dysplasia. HRIs undergo an annual screening that includes magnetic resonance imaging (MRI) and/or echoendoscopy (EUS), with additional blood based tests for the detection of tumor biomarkers associated with the pancreatic cancer. The Spanish registry of familial pancreatic cancer, PANGENFAM, was established in 2009 with the main objective of characterising the phenotype and genotype of FPC [20]. Within our screening program we have detected and successfully treated 4 malignant lesions (3 early PDAC and one neuroendocrine tumor) and together with two other international registries (Leiden and Marburg), we have critically analyzed the follow-up protocol to make it more efficient and cost-effective [21, 22]. Follow-up of HRIs is effective and improves early detection of the disease. However, the genetic basis of FPC is unknown in the majority of families, thus, all HRIs in the family are included in screening programs, even though approximately 50% will not be carriers of pathogenic germline mutations. Thus, there is a need to identify true HRIs by other techniques, without knowing the specific mutation within the family. The Best Linear Unbiased Prediction (BLUP) is used to study complex traits and allows the prediction of individual genetic risk. Using BLUP, the components of phenotypic variance can be estimated to determine heritability, i.e. whether cases of cancer within the same family are actually due to a heritable factor or whether they are the product of shared environmental factors.

The objective of this study was to estimate the value of the heritability of PDAC, based on the hypothesis that there is an additive genetic component and that an additive genetic component exists. We have previously shown that BLUP can be used to obtain a reliable estimation of heritability for cancer using the Minnesota Breast Cancer data set [23]. BLUP uses a mixed model to simulate individual risk of developing cancer, considering fixed and other variable factors within a population. Three models were used, combining different fixed effects (sex + family + generation) and random effects (individual + family + generation) to estimate the heritability of pancreatic cancer and the individual genetic risk of developing cancer. The main aim was to use this value as a follow-up criterion in FPC families, where the genetic basis of the syndrome is unknown. This analysis could complement the on-going phenotypic, molecular and imaging characterization of the HRIs, to further optimize the screening program, reducing the associated psychological and economic burden.

## Materials and methods

### PANGENFAM inclusion criteria and data collection

Inclusion criteria: (1) FPC families with ≥ 2 affected first or second degree relatives; (2) Hereditary breast and ovarian cancer (HBOC) families with at least one case of PDAC; (3) Families with ATM mutation and at least one case of PDAC; (4) Familial atypical multiple mole melanoma (FAMMM) families with at least one case of PDAC; (5) Hereditary Non Polyposis Colorectal Cancer (HNPCC) or Lynch Syndrome families with at least one case of PDAC; (6) Peutz Jeghers families; (7) Hereditary Pancreatitis (with pathogenic variants in the genes PRSS1 and SPINK1); and (8) Families with PDAC cases diagnosed at ≤ 50 years of age [20].

Some high-risk individuals underwent routine genetic testing in the clinic for known familial cancer associated genes, including BRCA2, BRCA1, CDKN2A, MLH1, ATM, PALB2, CHEK2 and SPINK. Of the 71 individuals tested, 21 (30%) were positive for a pathogenic variant, most frequently in the BRCA2 gene (57%). The data for the study are stored in a secure sever in a custom designed database in REDCap (13.4.11, 2024 Vanderbilt University). The database used for this study was downloaded on 5 October 2022 and consisted of 4602 individuals from 125 families. Each family has a 9-digit unique identifier, and each individual has a unique 12-digit identifier. The kinship of each individual was used to construct the correlation matrix based on the offspring relationship and off-kindred individuals were excluded. Information available for each individual on clinical history, sex, age, family identifier and generation were selected to estimate variance components and define heritability. The first phase of the analysis included 3780 individuals, 752 cases of any cancer, of which 213 correspond to PDAC.

### Generation of the BLUP mixed model

Data from the families within PANGENFAM were used, assuming a genetic component, although the calculated risk encompassed all cancers with a possible genetic component and also those specifically with PDAC. First, we estimated the heritability of all cancer types with the information PANGENFAM pedigrees. The dependent variable was cancer yes or cancer no. Three models were used, adding sequentially a random effect, model I (individual), II (individual + family) and III (individual + family + generation), to which effects were added, taking as a reference the models described in our previous study that used the same model [23]. Model I was a very simple and biologically implausible model and Model III was more biologically plausible. The components of the 3 models are summarised in Table 1.

**Table 1:** Summary of the 3 mixed models, specifying the fixed and random effects in each case

Model	Fixed effects	Random effects
1 → I	Sex + family + generation	Individual
2 → II	Sex + generation	Individual + family
3 → III	Sex	Individual + family + generation

Subsequently, the dependent variable was refined using just PDAC, yes or no, and applying model III. In this way, the BLUP analysis was used (1) to estimate the heritability of pancreatic cancer and (2) estimate the individual genetic risk of developing pancreatic cancer. The software RStudio [24] was used and several specific packages were employed for further analysis including, “kinship2“ [25], “pedrigreemm” [26], “pedigree” to plot the family trees of each family, the package “MCMCglmm“ [27] for the calculation of the mixed model and the ROCR package [28] to analyse the predictive character of the model obtained.

### Statistical methodology for assessing individual risk of developing cancer

For this study, cancer was defined as a phenotypic trait resulting from the additive effect of a large number of genes with a medium-low effect, which supports the hypothesis that this disease has a heritable genetic component. The variable representing the cancer is binary, where a value of 1 is assigned to affected individuals and 0 to unaffected individuals. The usual model to study binary traits is a threshold model. This model assumes a continuous underlying random variable, liability, which when it is over a given threshold, this triggers the expression of one of the binary phenotypes, i.e. cancer or no cancer, and PDAC or no PDAC [29, 30]. The variance for this underlaying normal distribution was set to 1.

For the estimation of risk, the BLUP was calculated using the equations of the Henderson mixed model [31] and the Fisher infinitesimal model [32]. Generalized Linear Mixed Models (glmm) are an extension of the generalized linear models that allow the inclusion of response variables of different distributions, such as binary [27]. The linear mixed model was defined as:

1 $$y=X\beta +Zu+e$$

where y is the observed phenotype, β and u are the fixed and random effects respectively, X and Z are matrices and e is the random error. The random effects follow a multivariate normal distribution MVN, u ∼ MV N (0, G) and e ∼ MV B(0, R) where G is the genetic covariance matrix and R the residual. Henderson proposes the following solution to the model:

2 $$\left[\begin{array}{cc}X ^{\prime}{R}^{-1}X& X ^{\prime}{R}^{-1}Z\\ Z ^{\prime}{R}^{-1}X& Z^{\prime}{R}^{-1}Z+{G}^{-1}\end{array}\right]\left[\begin{array}{c}\widehat{\beta }\\ \widehat{u}\end{array}\right]=\left[\begin{array}{c}X^{\prime}{R}^{-1}y\\ Z^{\prime}{R}^{-1}y\end{array}\right]$$

The Fisher model states that genetic inheritance is based on an infinite number of loci with a small additive effect. The phenotypic variance VF is calculated as the sum of the genotypic variance VG and the environmental variance VE. In turn, the genetic variance is the sum of an additive component VA and a non-additive component VNA, related to dominance or epistasis effects. BLUP allows the calculation of the additive part of this heritability that is transmitted between generations. The heritability is calculated using Fisher’s expression:

3 $${h}^{2}=\frac{{V}_{A}}{{V}_{F}}$$

The following formula was used to estimate the heritability from the components of the calculation of model III:

4 $${h}^{2}=\frac{{\theta }_{individual}^{2}}{{\theta }_{individual}^{2}+{\theta }_{family}^{2}+{\theta }_{generation}^{2}}$$

Where $\theta$^2^, is the variance. Denominator of formula (4) is the phenotypic variance decompose on their components, given that we are using a threshold model. Numerator of formula (4) is the additive component of the variance attributable to individual. The consistency of this estimate of h^2^ was assessed by testing the null hypothesis of heritability, i.e. h^2^ = 0 using a Bayes factor. The input parameters for the calculation include the kinship matrix, which establishes the relationships between individuals, and a matrix with the values of each individual for each of the effects included in the models. For the calculation of each of these variances necessary for the estimation, Bayesian inference was used because it is a stochastic calculation using a binary variable. The models were run with 5.25 million iterations, with an initial burn-in of 250 000 iterations and sampling every 2500 iterations, resulting in a sample size of 2000. When the model was run for only PDAC as a dependent variable, a model with 6.25 million iterations, with the same burn-in and thinning interval as previous models lead to a sample size of 2400.

For the a priori distribution, an inverse-gamma distribution with parameter expansion was used, which is that recommended by the author of the calculation package for small variances as indicated in the manual [33]. The residual variance was set to θ^2^ = 1. Once the models were calculated, an analysis of the convergence of the Markov chain was carried out using the Heidelberg and Welch test [34] to reject or fail to reject the results obtained in the model calculation.

### Estimated genetic value calculation

The estimated genetic value (EGV) was obtained as the solution of the individual random effect, by calculating the mean of the 2000 samples obtained in the calculation of the variance associated with each one. The sampling interval chosen was sufficiently wide to reduce autocorrelation. Furthermore, to identify individuals at high risk for cancer based solely on family pedigree information, the individual risk was calculated as the mean of the values of their parents. This is because each individual inherits half of his additive genetic component from the father and half from the mother [31]. Pearson´s correlation coefficient was used to assess the correlation between family incidence of cancer and median EGV of the family. In order to assess differences in EGVs between groups non –parametric Kruskall Wallis test was used.

To assess the predictive ability of this calculation, the area under the Receiver Operating Characteristic (ROC) curve was used to assess the predictive ability of this calculation. Analysis of the ROC curve allows to distinguish between positive and negative cases during prediction, so that an area under the AUC (Area Under Curve) close to 1 indicates that the model has a high predictive ability to identify individuals at risk of developing cancer.

## Results

### Description of families

**Fig. 1:**
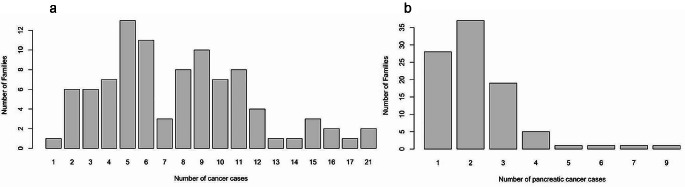
(**a**). Histogram of any cancer prevalence within the families and (**b**) pancreatic cancer prevalence within the families

After the cleaning and selection of the families of interest for the study, 96 families consisting of 4578 individuals were used for the final analysis. The remaining families did not meet the criteria for familial or hereditary pancreatic cancer or had data from few individuals available. For each individual, their cancer status was stored, which allowed the calculation of the incidence within the population, obtaining values for females of 0.154 and for males of 0.176. No significant differences were observed according to gender. Regarding the distribution of cancer within the families, each family consisted of 8 to 158 individuals, with between 1 and 22 reported cases of cancer (Fig. 1a) and 1–8 reported cases of PDAC (Fig. 1b). However, only families with 20 or more individuals were considered for the prevalence calculation because a smaller number tends to overestimate the prevalence and 16 families were excluded from this analysis. It is likely that these families were incomplete or that only the family branches and generations most affected by cancer were stored in the study database.

### Quality control of the data and EGV model

**Fig. 2:**
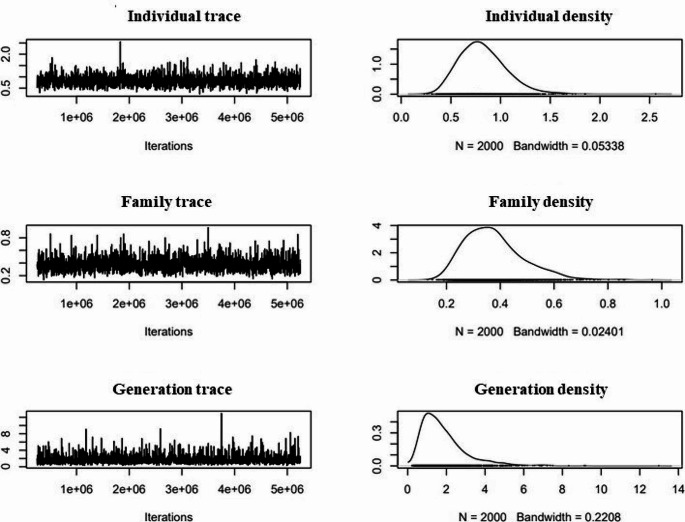
MCMC traces of variance (corresponding to VA) and density due to individual, family and generation for model III

The generation of the genealogical trees was used to verify the quality of the data, as it was a visual way to confirm the correct structure of the data, specifically, ensuring that the kinship matrix was correct, which guaranteed the validity of the model used in the study. Figure 2 shows the trace of model III and the subsequent density obtained for each calculated parameter. The Heidelberg test was applied to each model, which confirmed their convergence. These results validated the model calculation, allowing the analysis to proceed. The high posterior density intervals calculated by MCMCglmm for the variance components in model III were [0.3825–1.265] for the individual, [0.187–0.5932] for the family and [0.3128–4.235] for the generation. From these values, the heritability was estimated, obtaining a heritability value of [0.3128–4.235] for the generation. From these values, the heritability was estimated obtaining a value of 0.44 with a high posterior density interval [0.3030–0.5752] for the PANGENFAM cohort. The value of the interval was not null so the null hypothesis was rejected and allowed us to affirm that there was an additive genetic component in the development of PDAC.

Models I and II also converged, however, they were discarded as they were far from the biological reality. When analysing model I, a heritability of 0.99 [0.9843–0.9921] was obtained (Table 2), implying that cancer depends only on its genetic component and that almost all individuals would suffer from the disease. Given that model I attributed too much variability to the genetic component (individual) and this is not biologically likely, new random effects were added to the model until a plausible value was reached. Model II estimated a heritability of 0.39 [0.28–0.52], a value closer to reality. Finally, the generation effect allowed to include a temporal component to the model and to finish adjusting the heritability value to 0.21 [0.1–0.33] in model III (Table 2). The more biologically plausible model III was also run for PDAC only as a more strict assessment of hereditability, with 500,000 and 600,000 iterations, giving a hereditary value of 0.27.

**Table 2:** Heritability estimates for different models using all cancer types or PDAC as the dependent variable. Real scale is the observable scale of the trait versus the liability scale which is non-observable. Liability follows a standardized normal distribution that after a certain threshold triggers the illness

Dependent variable	Heritability	Liability scale	Real scale
All cancer types	Model I	0.99 [0.98–0.99]	0.55 [0.41–0.63]
	Model II	0.39 [0.28–0.52]	0.22 [0.15–0.29]
	Model III	0.21 [0.1–0.33]	0.1 [0.04–0.16]
Only PDAC (Model III)	500,000 iterations	0.27 [0.06–0.47]	0.07 [0.02–0.12]
Only PDAC (Model III)	600,000 iterations	0.27 [0.07–0.47]	0.07 [0.02–0.13]

**Fig. 3:**
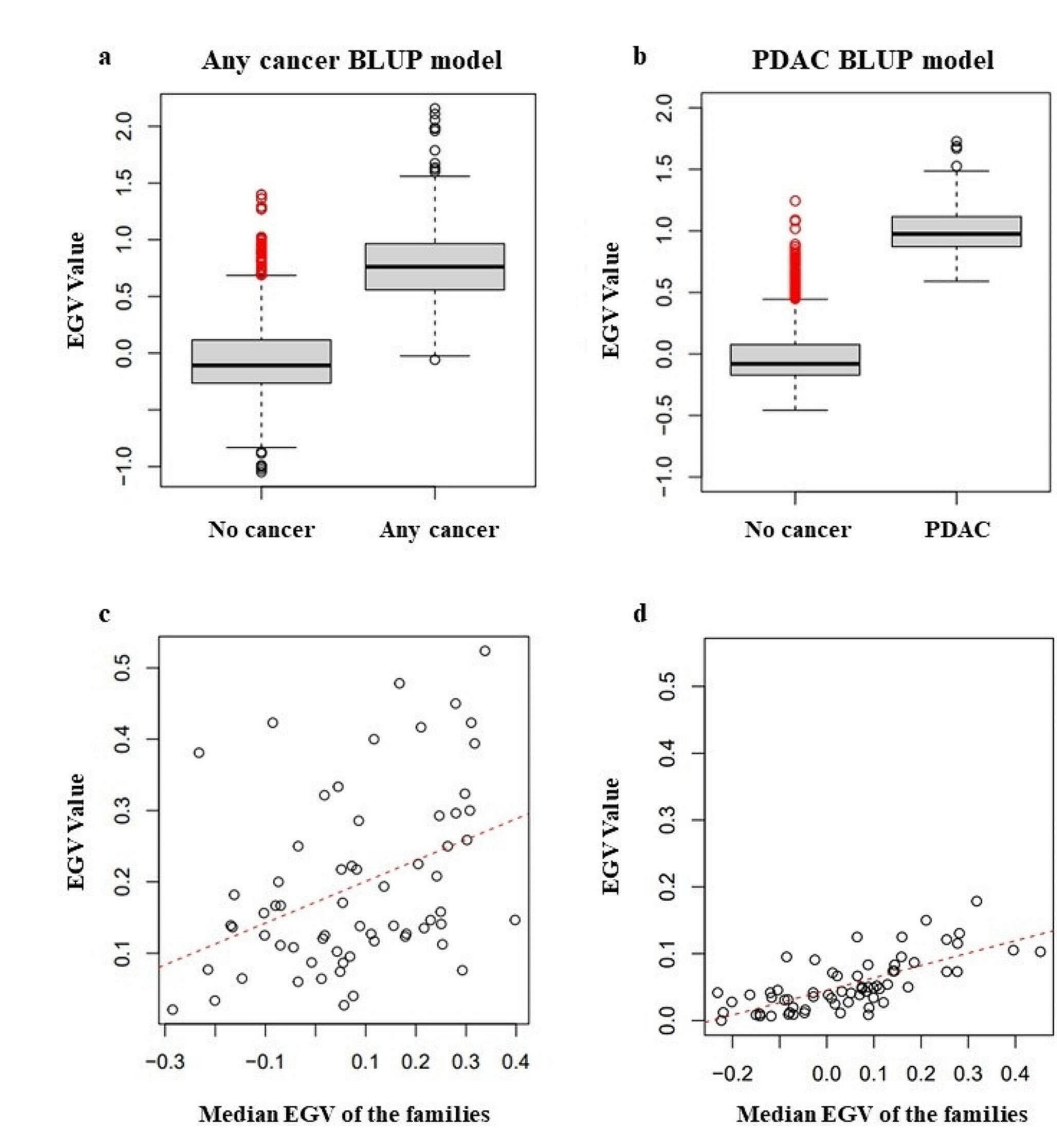
(**a**) Boxplot of comparison of EGVs according to the any cancer BLUP model and (**b**) PDAC BLUP model; 0 = no cancer, 1 = any cancer. (**c**) Correlation between family prevalence and median EGV of the family according to the any cancer BLUP model (Pearson´s correlation coefficient 0.65 [0.5–0.76]; p value < 0.001) and PDAC BLUP model (Pearson´s correlation coefficient 0.61 [0.45–0.73]; p value < 0.001)

PDAC heritability was estimated only using Model III. The EGV is a measure of the additive genetic risk of developing cancer, so individuals without cancer are expected to have a lower EGV than those who developed the disease. Figure 3a shows that the EGV (with corresponding interquartile range (IQV) using the *any cancer BLUP model* is significantly higher for individuals with any cancer vs. no cancer (no cancer − 0.108 IQR [-0.264;0.116] vs. any cancer 0.762 IQR [0.558;0.966], p-value < 0.001). Furthermore, the EGV using the *PDAC BLUP model* was significantly higher for individuals with PDAC versus no PDAC, (no PDAC − 0.081 IQR [-0.172;0.075] vs. PDAC 0.974 IQR [0.870;1.116], p-value < 0.001). Subsequent analyses of EGV was performed using the 2 models, the any cancer BLUP model based on individuals with any cancer vs. no cancer and, the more strict PDAC BLUP model based on individuals with PDAC versus no PDAC. For the screening programme, it is of interest to identify individuals with high EGV who have not yet developed the disease (shown in red in Fig. 3a and b). It is also expected that families with a higher prevalence will have a higher mean EGV, assuming a higher genetic predisposition. Figure 3c and d identifies some relationship, although many families deviate from the trend. This is because the prevalence cannot exceed 0.5 in any case despite having a high EGV, since not all individuals carry the risk mutations.

**Fig. 4:**
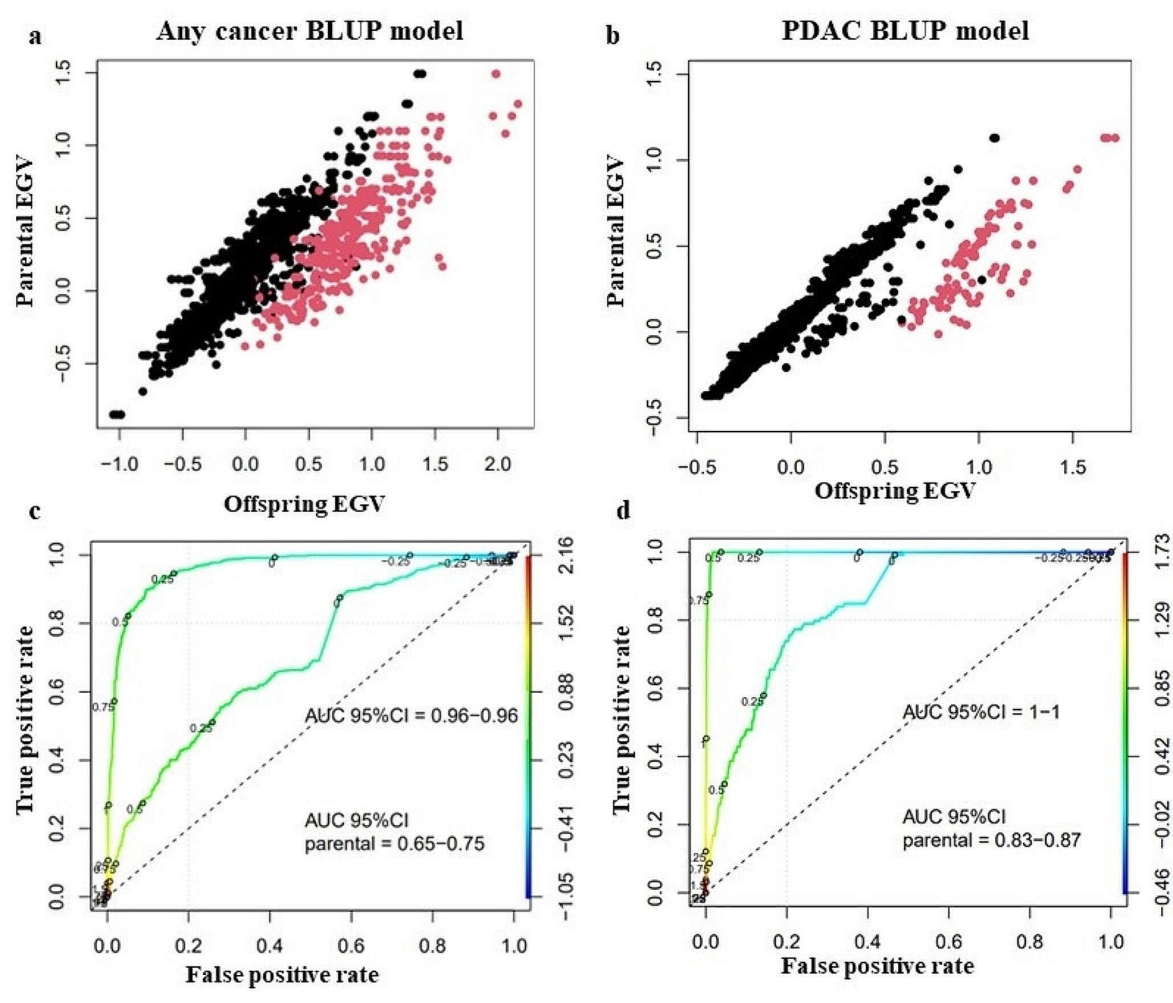
Average EGV of parents versus calculated EGV of offspring, individuals with cancer are shown in red and individuals without cancer are shown in black, (**a**) using the any cancer BLUP model and (**b**) using the PDAC BLUP model. ROC curve using the mean EGV of the parents (**c**) for the any cancer BLUP model and (**d**) for the PDAC BLUP model. *EGV was used as a cancer predictor, using either the EGV value assigned to the individual or the parental mean of the EGV. Both ROC curves have an AUC above 0.5*

An individual inherits half of the genetic load of each of its parents, and an individual’s EGV can be estimated as the mean of the EGV of the parents and used as a predictive model for new generations. Figure 4a shows the results of the calculation of the mean EGV, showing that individuals with any cancer using the general model had a higher mean EGV than individuals without cancer; (Pearson´s correlation coefficient 0.76 [0.74–0.78] p value < 0.001). Using the more strict model PDAC BLUP model of no PDAC vs. PDAC, individuals with PDAC had a higher mean EGV than individuals without PDAC; (Pearson´s correlation coefficient 0.85 [0.84–0.86] p value < 0.001) (Fig. 4b). In Fig. 4c, the ROC curve allowed the predictive character of the estimate of EGV to be evaluated, as the mean of the parents compared to the cancer status. A reasonably high AUC of [0.65–0.75] was obtained when using the mean of the parents and 0.96 for the any cancer BLUP model. However, an AUC of [0.83–0.87] was obtained with this model (Fig. 4d), indicating that an individual with a high positive EGV has a high genetic predisposition to develop PDAC. Thus this model was used for all subsequent analysis.

**Fig. 5:**
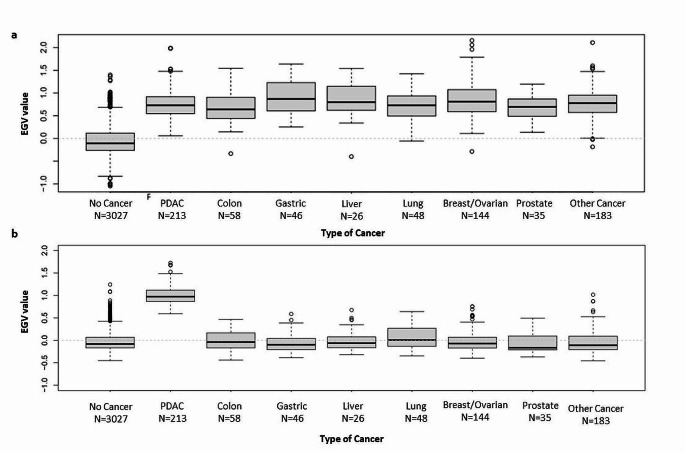
Comparison of the EGV in individuals with different types of cancer and individuals without cancer, including the number of individuals in each group using the (**a**) Any cancer BLUP model and (**b**) PDAC BLUP model

The EGV was significantly higher for cases with PDAC compared with individuals without cancer or PDAC; any cancer BLUP model: no cancer/no-PDAC − 0.108 IQR [-0.264;0.116] vs. PDAC/no PDAC 0.729 IQR [0.547;0.918], (p-value < 0.001) (Fig. 5a), and PDAC BLUP model: no PDAC − 0.082 IQR [-0.169;0.069] vs. PDAC 0.974 IQR [0.870;1.117] (p-value < 0.001) (Fig. 5b). The outliers in the “no cancer” group shown in Fig. 5b are those individuals who theoretically should be followed more intensively periodically, as they are at higher risk of developing PDAC. Furthermore, there are some outliers within the group of individuals with breast and ovarian cancer as well as the “other cancer” group, which may also have a hereditary background. The EGV of other tumor types are around or below zero as they most likely related to sporadic cases that are attributed to certain environmental factors, such as smoking in the case of lung cancer.

### EGV of patients on follow-up

**Fig. 6:**
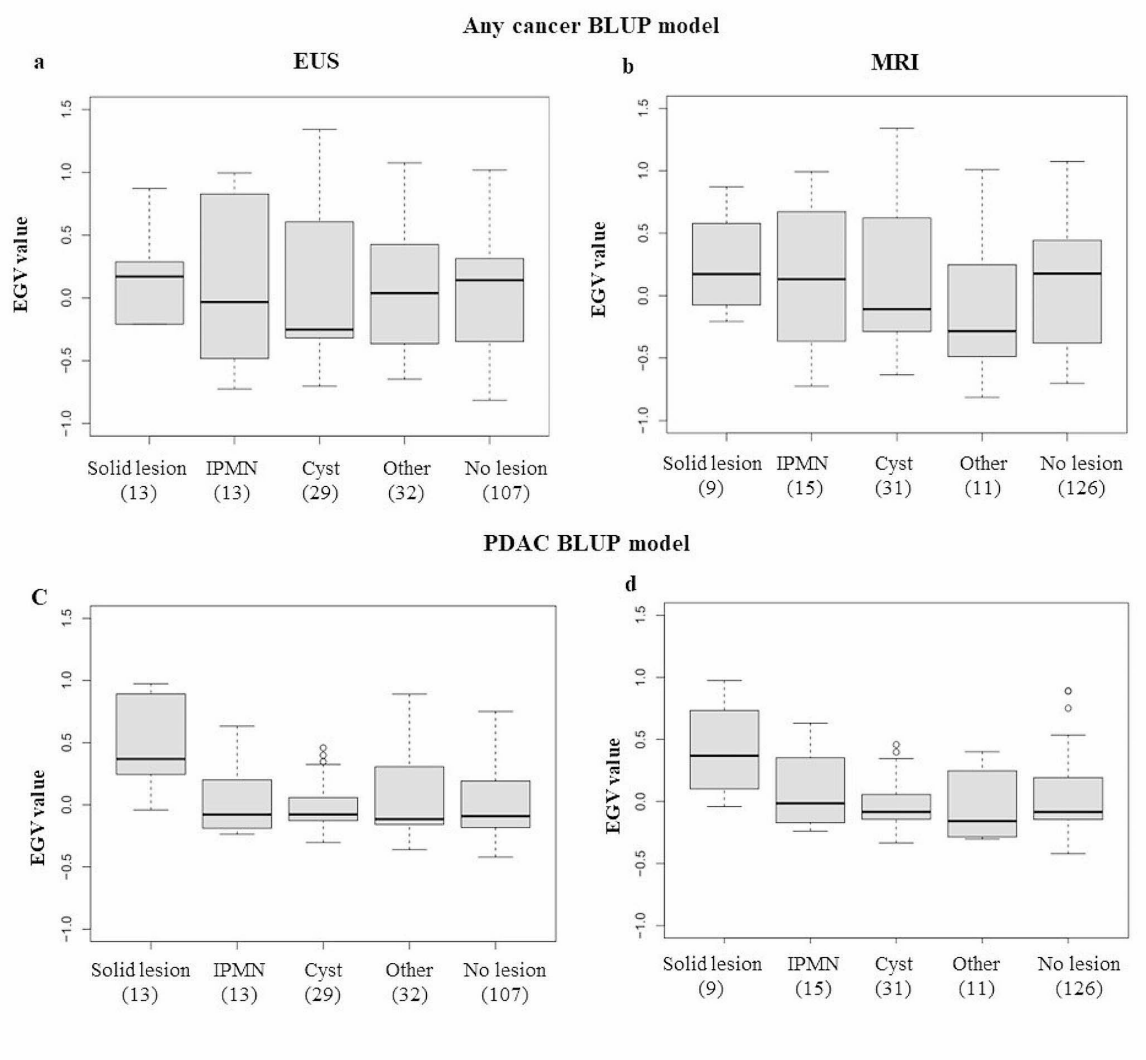
Comparison of EGV in individuals with different types of lesions detected during follow-up as a function of the detection technique used, according to the no cancer-any cancer BLUP model by (**a**) EUS and (**b**) MRI and the PDAC BLUP model by (**c**) EUS and (**d**) MRI

High-risk individuals in follow-up undergo annual MRI and EUS screening, which identifies lesions within the pancreas, as well as outside of the pancreas in the case of MRI. Figure 6 shows the study of the relationship between the EGV of the individuals with the type of pancreatic lesion identified by MRI and EUS and their corresponding age. Figure 6a shows the lesions detected by EUS were and Fig. 6B shows the lesions identified by MRI. Solid lesions had a positive mean EGV (any cancer BLUP model: EUS 0.170 IQR [-0.141;0.285], MRI 0.173 IQR [-0.008;0.532], PDAC BLUP model: EUS 0.369 IQR [0.244;0.792], MRI 0.369 [0.173;0.613]), independently of the screening modality used, as expected. Whereas, pancreatic cysts, IPMN and other lesions (mainly inhomogeneous pancreas parenchyma) had a mean EGV around or lower than zero, similar to the mean EGV in individuals with no pancreatic lesions. Cysts are generally benign or have a low probability of malignant progression. Interestingly, individuals with IPMNs detected by MRI (Fig. 6b) had slightly higher mean EGV (any cancer BLUP model: 0.131 IQR [-0.365;0.673], PDAC BLUP model: -0.014 IQR [-0.171;0.352]), lower than the mean EGV of individuals with solid lesions and slightly higher than the mean EGV for individuals with cysts and no pancreas lesion, although this did not reach statistical significance. This is logical as IPMN are considered as precursor lesions of PDAC, with a variable risk of malignant progression. Interestingly, there were some outliers with high EGVs in the cyst cohort detected by both EUS and MRI and in the no lesion cohort. This is also consistent with the theory that some of the individuals with no solid pancreatic lesions have a potential future diagnosis of PDAC or its precursor lesions. There was no significant difference in age at diagnosis of the different types of pancreatic lesions, although those with IPMN were slightly older compared with those diagnosed with cysts. Furthermore, those with normal pancreatic imaging were slightly older than those with other types of pancreatic lesions.

**Fig. 7:**
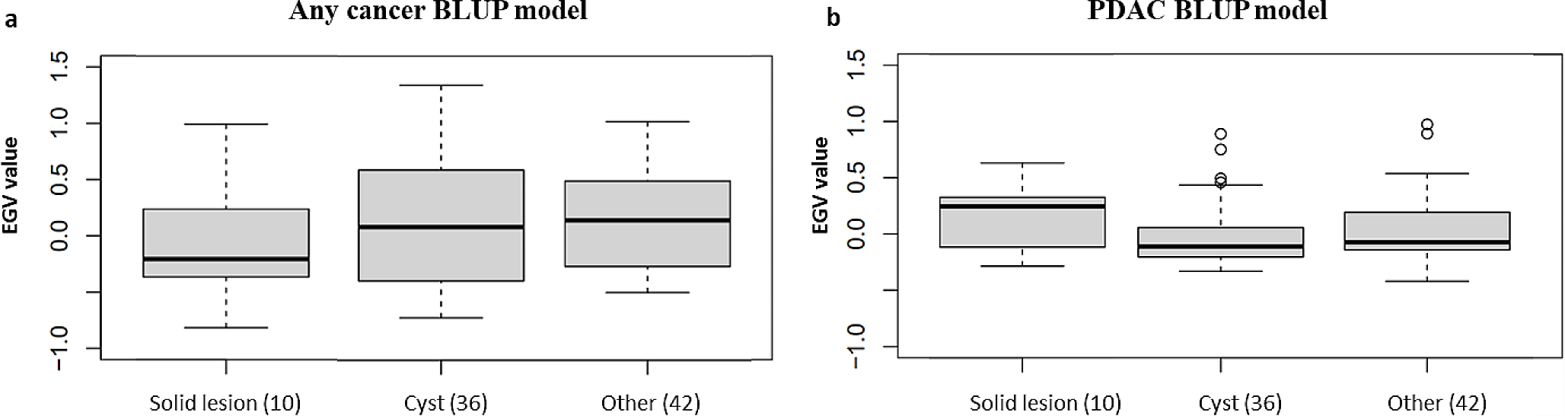
Comparison of EGV in individuals with different types of extra-pancreatic lesions detected on MRI and EUS. (**a**) No cancer-any cancer model (**b**) No pancreatic cancer-pancreatic cancer model

Finally, the relationship of EGV with other types of extra-pancreatic lesions detected during patient follow-up was analysed. Figure 7 shows that individuals with non-pancreatic solid lesions have mean EGV below zero, this is logical as these solid lesions are not expected to be related to familial PDAC risk. The majority of extra-pancreatic lesions were mainly liver and renal cysts, and these individuals had EGV approaching zero. Whereas, the mean EGV for individuals with other extra-pancreatic lesions was slightly higher; any cancer BLUP model: 0.141 IQR [-0.261;0.477] (Fig. 7a), PDAC BLUP model: -0.073 IQR [-0.141;0.192] (Fig. 7b), but did not approach those seen in individuals with solid pancreatic lesions.

## Discussion

The heritability estimated by BLUP calculation in this study of families with familial pancreatic cancer differs from 0, confirming the hypothesis of polygeny in the inheritance pattern of this disease. EGV allow distinguishing between individuals with cancer and those without the disease, which makes it a useful tool to estimate the cancer risk of an individual and to include them in a high-risk screening programme. In this case, the heritability was estimated at 0.44, a value similar to that obtained in our previous study [23], where a heritability of breast cancer was estimated at [0.017–0.396]. In theory, individuals with a negative EGV are considered to be free of genetic risk for cancer. However, this does not rule out the possibility that they may develop the disease due to the action of other factors such as environmental factors. On the other hand, individuals with a positive EGV have a hereditary basis that predisposes them to the disease to a greater or lesser extent, depending on the value.

The estimated heritability for other complex diseases is 52% for Amyotrophic Lateral Sclerosis [35], 17–21% for schizophrenia [36] and a h2 = 0.67–0.91 for the form of the hippocampal subregion that predisposes to Alzheimer’s disease [37]. In comparison, the heritability value of the pancreas is moderate, with less than half of the variation in disease susceptibility attributable to genetic factors. However, pedigree-based risk estimation is not a widespread procedure and predictive models with a genetic basis and specific mutations are often chosen. As an example, the Gail model is used in breast cancer prediction [38], as shown in the study by Johansson et al. [39] where an AUC of 61.8% is obtained using this logistic regression model incorporating 10 genetic variants. Another example is the prediction model of Wu et al. [40], which obtained an AUC of 80% for the prostate cancer using a similar methodology. Also noteworthy is the study by Lee et al. [41], which uses the predictive model BOADICEA (Breast and Ovarian Analysis of Disease Prevalence and Carrier Estimation Algorithm) which combines high-risk mutations with other associated environmental factors, and shows that the best possible prediction of breast cancer is achieved by combining both genetic and environmental factors. Comparing these values to those obtained using the BLUP calculation, where an AUC of 95% was achieved for the individual EGV estimated by the model and an AUC of 65–75% when the prediction was performed using the mean of EGV of the parents, the latter is at the same predictive level as the previous models, being equally powerful despite not considering specific mutations in the calculation.

In the case of PDAC, current research is focused next generation genomic analysis to identify variants associated with the occurrence of the disease. Several Polygenic Risk Scores (PRS) models for PDAC have been developed, including 4 models that include 5, 30, 33 and 22 SNPs [42–45], respectively, that predispose to the development of PDAC. However, these studies are costly as sequencing is still an expensive technique. Moreover, the clinical application of these techniques has not yet been achieved and there are doubts about their efficacy [46, 47]. Therefore, it is important to highlight the relevance of studies with models such as BLUP, as they only require a computer and a database to identify individuals with a potential risk of developing PDAC. These models can be incorporated in hospitals at no great additional cost. In addition, they allow the study of a large number of patients and, as they are specific to each individual, they provide a more personalised, faster and more effective follow-up.

The mean EGV for individuals with solid extra-pancreatic lesions was much lower than the in individuals with solid pancreatic lesions. Thus, this supports the notion that the BLUP model can predict PDAC risk, as it does not assign a high EGV in the presence of non-PDAC solid lesions in high risk individuals, although a small proportion of these lesions may progress to PDAC. The reported surgical intervention rates due to suspicious lesions in this population type is around 5% [48–50], and specifically 2.6% of individuals from our registry (REFERENCE). To achieve a high-degree of reliability, the BLUP model requires good quality multi generation pedigree information and a large of number of pedigrees with accurate information of cancer diagnosis. The calculation of the EGV can be made more accurate by adding more effects to the model, such as clinical, sociological, or demographic characteristics. However, obtaining these data for the entire population is more difficult. Nevertheless, it would be possible to add the associated pathogenic variants as they are discovered. Another way to improve the BLUP model would be to incorporate genetic information in the kinship matrix, in the form of coefficients to estimate genetic values. The genetic values could be estimated by combining phenotypic, pedigree and genomic information at the same time [51].

At present, early diagnosis and the development of new, truly effective therapeutic strategies for the treatment of pancreatic cancer remain essential. Widespread screening at the population level is not feasible due to the low prevalence of this type of cancer and the invasive techniques used to detect pancreatic lesions. It is only justified in defined high-risk populations, such as those with hereditary or familial pancreatic cancer. Therefore, new specific, sensitive and minimally invasive approaches are needed, and the calculation of EGV for HRI can serve as a starting point to predict the risk of this deadly disease. However, in order for algorithms such as these to be accurate and useful assessments for the management of high-risk individuals, we must ensure that input data is of high-quality, including pedigree information, full medical history and epidemiological data. Importantly, there are several international consortiums that provide guidelines and recommendations for high-risk screening [16–19], as well as templates for standardized reporting and interpretation of the EUS [52] and MRI [53] imaging tests.

## Conclusions

The BLUP model offers a valuable tool for the study of hereditary cancer, allowing to estimate the degree of heritability of the cancer and to calculate the individual genetic risk in the members of a family with a history, as well as an approximation to the genetic risk in future generations. In addition, this tool can be used to identify the likely carriers of pathogenic variants, that should be prioritized for next-generation sequencing analysis. As a next step in the validation of the model, it is suggested to apply it to a larger population, standardising the data of the families involved.

## Data Availability

No datasets were generated or analysed during the current study.
